# C^2^RQA as the quantification method of the dynamics of collaborative creativity

**DOI:** 10.3389/fpsyg.2026.1825028

**Published:** 2026-09-01

**Authors:** Daichi Shimizu, Takeshi Okada

**Affiliations:** 1 Graduate School of Human Development and Environment, Kobe University, Kobe, Hyogo, Japan; 2 Graduate School of Education, The University of Tokyo, Tokyo, Japan

**Keywords:** collaborative creativity, cross recurrence quantification analysis, interaction process, quantification, visualization

## Abstract

This study introduces Content-based Cross-Recurrence Quantification Analysis (C^2^RQA) as a useful approach for visualizing and quantifying the dynamics of collaborative creative processes. Although the significance of the creative process is widely recognized in the existing literature, its inherent complexities—such as emergence, revisitation, representation, embodiment, and the interplay between conscious and subconscious elements—have historically hindered the development of standardized quantitative assessments. As a step toward addressing this gap, we propose Content-based Cross-Recurrence Quantification Analysis (C^2^RQA), an extension of CRQA that can process continuous, categorical, and binary data. By mapping detailed content correspondences between two time series, C^2^RQA enables examination of the complexity and dynamics of creative interactions. Our application of this method to idea generation, insight problem-solving, and drawing activities in art workshops illustrates its potential for capturing both macro-level conceptual transitions and micro-level cognitive and behavioral processes involved in collective creative endeavors.

## 1 Introduction

The present study presents a novel analytical framework designed to visualize and quantify the dynamics of multi-individual creative processes. We illustrate the proposed approach through its application to three experimental and field datasets representing different types of creative activities. Established literature on creativity has long emphasized the significance of the creative process, along with elements such as person, product, and press in Rhodes’ (1961) 4P’s model. Contemporary scholarship further advocates an emergent perspective, suggesting that creative outcomes emerge from the continuous process of interaction among creators, their collaborators, and the environmental context (e.g., Glăveanu, 2013; Okada and Yokochi, 2025; Sawyer and DeZutter, 2009).

To develop an effective analytical lens on the creative process, we must identify its defining properties, particularly within short-term sessions lasting minutes to hours. Building on prior studies and synthesizing their conceptual perspectives, we identify three fundamental dimensions:

Emergence and Revisitation: Creative endeavors are characterized as ill-structured problem-solving where solutions are not predetermined (Klahr and Dunbar, 1988; Simon and Newell, 1971). Consequently, ideas are continuously generated, developed, and reactivated through the recursive cycle of generative and exploratory phases (Finke et al., 1996). Further, several studies empirically demonstrated that the ideas initially dismissed may be revitalized through new insights, a non-linear cycle observed in both experimental divergent thinking tasks and naturalistic artistic and design production (Botella et al., 2013; Jaarsveld and van Leeuwen, 2005; Okada et al., 2009; Shimizu and Okada, 2018). This cycle of emergence and revisitation, with revisitation representing a concept synthesized from prior studies, corresponds to key aspects of the idea-generation and exploration process described in those studies.

Representation and Embodiment: As in scientific discovery, individuals engage in higher-order, conceptual thinking using words and images during creative activities (Dunbar, 1993; Okada and Simon, 1997; Simon and Newell, 1971). However, it is also deeply rooted in the physical body and its surroundings. For example, the discovery of concepts in mathematics often involves physical shifts in proximity to work surfaces (whiteboard) at critical moments of insight (Tabatabaeian et al., 2024). Similarly, studies of artistic creation show that cognitive processes, bodily actions, and the perceptual experiences linking them dynamically interact during the production of artwork (Botella et al., 2013; Okada and Yokochi, 2025; Shimizu et al., 2019; Yokochi and Okada, 2005). These findings illustrate how creative processes emerge through complex, active interactions between mental representation and the body.

Conscious and Subconscious Interplay: Traditionally, creative ideas and products have been viewed as outcomes of higher-order, conscious thought (Klahr and Dunbar, 1993; Simon and Newell, 1971). However, in artistic domains such as painting and dance, unconsciously produced lines or movements—those made without clear intention—can significantly shape subsequent conscious actions (Ross and Vallée-Tourangeau, 2021; Shimizu and Okada, 2018; Okada and Yokochi, 2025). Similarly, qualitative studies of theatrical performance show that unintended utterances or gestures can be reinterpreted by others in subsequent interactions, and through this accumulation, the narrative’s meaning gradually emerges (Sawyer and DeZutter, 2009). While the notion of “creator’s intention” requires further conceptual clarification, these findings suggest that creativity unfolds through interactions between intentional and unintentional utterance and action.

Considering these fundamental characteristics of creative engagement—namely, its emergent and iterative nature, its conceptual and embodied basis, and the synergy between conscious and subconscious forces—it is important to trace the longitudinal path of idea evolution. Achieving this ultimately requires a holistic analytical perspective that can incorporate multimodal inputs, such as images, linguistic, and action data, while accounting for both intentional and non-conscious aspects. Although the proposed framework was motivated by these broader theoretical perspectives, the present study primarily focuses on emergence and revisitation processes. The framework is demonstrated across multiple modalities, but crossmodal interactions and conscious/subconscious processes were not explicitly examined and remain important directions for future research. Likewise, although behavioral data were analyzed, the embodied mechanisms underlying creative interaction warrant further investigation.

This study specifically focuses on collaborative creativity, in which the aforementioned features are particularly salient. Previous studies have demonstrated how ideas evolve through inter-individual conceptual and embodied interaction (e.g., Dunbar, 1999; Okada and Simon, 1997; Sawyer and DeZutter, 2009). However, existing research has often adopted a “snapshot” approach, focusing on peak moments of insight rather than the longitudinal trajectory of creative activity. There remains a notable lack of bottom-up methodologies capable of integrating multiple modalities to track the unfolding creative process over time. To bridge this methodological gap, we propose an analytical framework for visualizing and quantifying the longitudinal dynamics of collaborative creative engagement. The proposed framework is primarily grounded in the principles of emergence and revisitation, whereas embodiment, conscious/subconscious interaction, and crossmodal integration provide broader theoretical motivations and represent important directions for future research rather than aspects directly examined in the present study.

## 2 Theoretical foundations of content-based cross recurrence quantification analysis (C^2^RQA)

### 2.1 Overview of cross recurrence quantification analysis (CRQA)

This section outlines the principles of Cross Recurrence Plot (CRP) and Cross Recurrence Quantification Analysis (CRQA), which serve as the structural basis for our proposed methodology. For a detailed mathematical treatment, which is beyond the scope of this paper, we refer readers to the seminal reviews by Marwan (2008) and Marwan and Webber Jr (2014).

**Figure 1:**
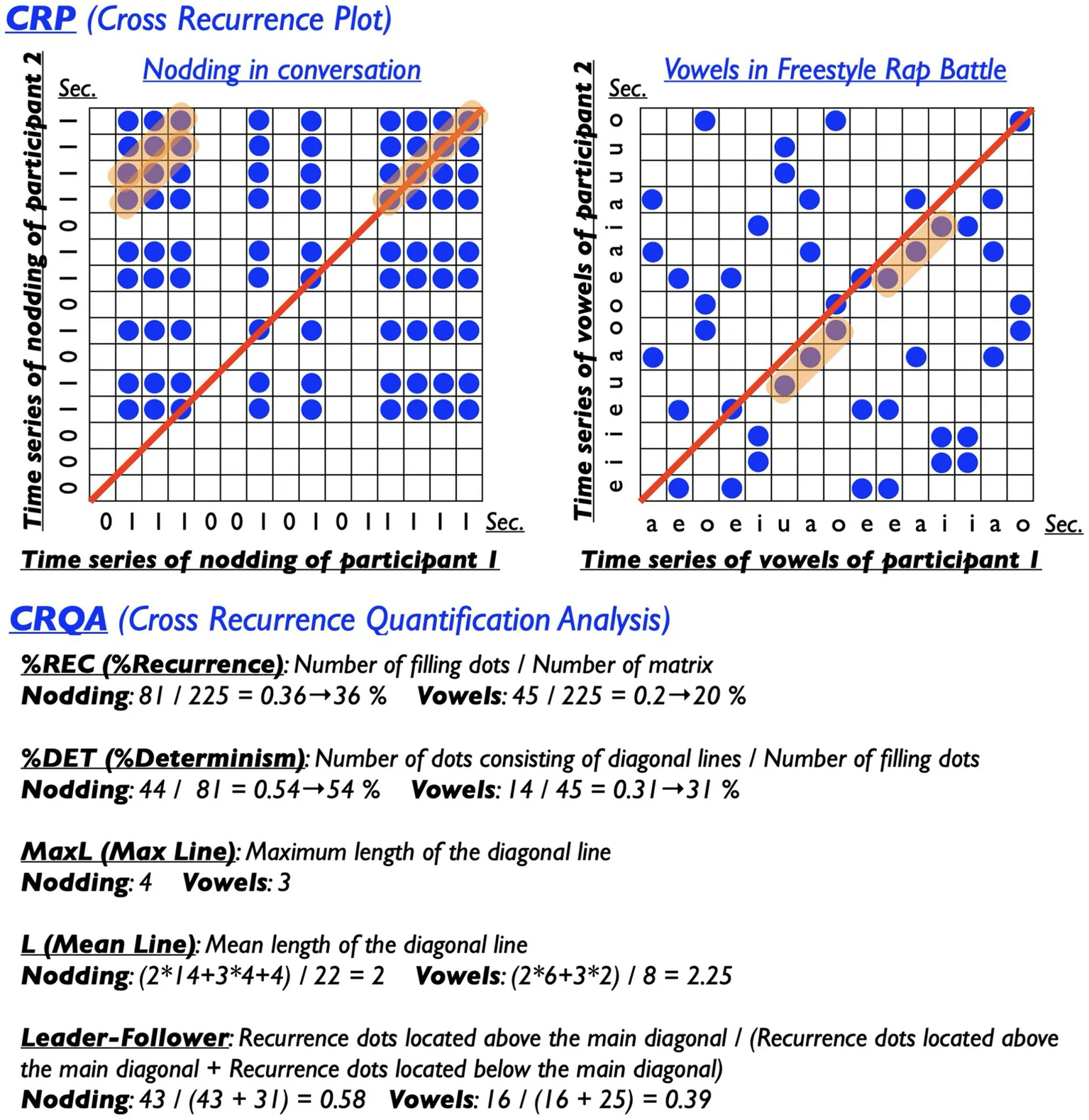
Illustrative examples of Cross Recurrence Plots (CRPs) and Cross-Recurrence Quantification Analysis (CRQA) generated from dummy datasets created by the authors to facilitate understanding of the CRP/CRQA framework. For visualization purposes, both examples depict only the first 15 s of activities that originally lasted several to more than 10 min. (Top) Examples of CRPs for two activities. The top-left panel shows an example of coordination in nodding behavior during a two-person conversation, “0” indicates the absence of nodding and “1” indicates the presence of nodding. The first example uses binary data because it provides the simplest and most intuitive illustration of how CRPs and CRQA visualize revisitation and coordination processes. The top-right panel shows an example of coordination in vowel usage during a two-person freestyle rap battle. The labels, “a, i, u, e, o,” indicate the vowels of each lyric. (Bottom) Examples of CRQA results derived from the corresponding CRPs shown above.

As an advancement of non-linear time series analysis, CRP and CRQA were designed to visualize and quantify the degree of synchronization and coordination between two distinct time series (cf. Marwan and Kurths, 2002; Zbilut et al., 1998). The analytical process involves mapping two time series onto the vertical and horizontal axes of a two-dimensional grid. For categorical data, the state space is reconstructed directly from time series data, whereas continuous data requires phase-space embedding of each time series. As this study focuses primarily on categorical data, discussions related to embedding are omitted (cf. Marwan, 2011). A Cross Recurrence Plot (CRP) is then generated by marking points where the states of the two systems align or achieve proximity, visually representing their temporal correspondence (see Figure 1, top). This graphical representation is referred to as a Cross Recurrence Plot (CRP).

### 2.2 Quantification metrics and historical context of CRQA

Subsequently, we introduce Cross-Recurrence Quantification Analysis (CRQA), a method for extracting quantitative measures from the Cross Recurrence Plot. CRQA primarily analyses various linear structures within the plot, with particular focus on diagonal lines. These diagonal trajectories indicate the sustained synchronization and coordination between the two time series. By evaluating the density and temporal extent of these lines, CRQA provides a quantitative measure of the dynamics and frequency of such interactions (Figure 1, bottom). A range of established metrics has been developed for this purpose (Fusaroli et al., 2014; Marwan and Kurths, 2002; Zbilut et al., 1998). Key indicators include %REC (Recurrence, capturing the overall level of system-wide coordination), %DET (Determinism, reflecting the ratio and duration of diagonal structures), MaxL (Maximum Line length, indicating the longest period of continuous coordination), L (Mean Line length, representing the average duration of coordination states), and ENT (Entropy, quantifying the structural complexity of the diagonal line distribution).

Originally conceived in the 1980s through the lens of nonlinear dynamics and chaos theory, the CRP/CRQA framework has matured through diverse applications in geosciences, economics, chemistry, and physics (Marwan, 2008; Marwan and Kraemer, 2023; Marwan and Webber Jr, 2014). From the early 2000s onward, its use expanded significantly across the behavioral sciences, facilitating the study of multi-agent interactions in physiology, psychology, and cognitive science. Empirical examples include the assessment of postural stability during verbal exchange (Shockley, 2005; Pellecchia and Shockley, 2005), gaze synchronization during collaborative Tangram tasks (Dale et al., 2011; Richardson and Dale, 2005; Richardson et al., 2007), hand movement coordination in rock-paper-scissors games (Kodama et al., 2019), and heart rate entrainment between performers and spectators in dance battle (Shimizu et al., 2024).

Consequently, CRP/CRQA has emerged as a versatile instrument for the visualization and statistical evaluation of interpersonal dynamics across numerous disciplines. This analysis enables researchers to capture the overall patterns of synchronization and coordination across a wide range of data types, including continuous, categorical, and binary data. In the present study, we refine and extend this methodology to adjust to the unique temporal properties of the creative process, utilizing it to analyze the cognitive and behavioral trajectories of participants involved in collective creativity.

### 2.3 Proposed extensions of CRQA for collaborative creativity

Building upon this foundation, this study introduces three specific extensions to CRP and CRQA, tailored to the unique demands of analyzing collaborative creativity. The original CRP/CRQA framework has several limitations for capturing key features of collaborative creative processes. First, all instances of synchronization and coordination are represented as equivalent states regardless of their content. Second, it does not sufficiently examine directional aspects of interaction, such as which participant introduced an idea first and how the other participant subsequently developed it. Third, it was not designed to readily integrate data from different modalities, such as verbal communication and bodily movement. In light of these considerations, we developed the following three extensions:

1) Semantic Color-Coding: To address the characteristics of emergence and revisitation, we introduced a color-coding system that categorizes recurrence points based on their conceptual content. While conventional CRP and CRQA typically employs a binary black-and-white scheme for all coordinated instances, this modification facilitates a more distinct visualization of when particular ideas are initiated, changed, and reactivated by multiple participants, thereby clarifying the transitions between ideational states.
2) Directional Dimension: Following established analytical strategies, we compared the density of recurrence points situated above and below the main diagonal of each color-coded utterance and action. This technique elucidates the directionality of interactions—specifically identifying which individual first proposed a specific concept and how it was subsequently elaborated upon by others. Although directionality is not explicitly defined as a primary feature of the creative process itself, it remains a fundamental component of collaborative dynamics.
3) Multimodal Integration: The analysis was expanded to encompass multiple modalities, integrating verbal discourse with physical movement. This multimodal approach, which builds upon existing research (e.g., Wallot and Leonardi, 2018), allows for the identification of patterns associated with representation and embodiment within the process of collaborative creativity.

Collectively, these extensions provide a promising means to visualize and quantify the intricate and flexible nature of joint creative efforts. In the following sections, we apply the proposed C^2^RP/C^2^RQA framework to several collaborative creative activities in order to examine its utility. It should be noted that the present study primarily provides empirical demonstrations of two extensions: (1) Semantic Color-Coding and (2) the Directional Dimension. In contrast, although (3) Although multimodal integration is theoretically supported and appears technically feasible based on our previous work (Kodama et al., 2021), it has not yet been explicitly examined within the present study. Consequently, the analyses presented here demonstrate the ability of the framework to capture aspects of the dimensions introduced earlier, particularly Emergence and Revisitation, and parts of Representation and Embodiment. However, whether the framework can effectively capture aspects of Conscious and Subconscious Interplay remains an open question requiring further empirical investigation. We return to this limitation in the discussion section.

## 3 Application of the analysis to diverse creative processes

### 3.1 Experimental dataset 1: Collaborative idea generation

This section demonstrates the application of the C^2^RQA framework to three prototypical creative tasks: idea generation, insight problem solving, and drawing activities in an art workshop. For the sake of brevity, specific experimental protocols are summarized; comprehensive procedural details are available in related literature.

We first examined a dyadic ideation task, a standard paradigm for investigating creative cognition (Finke et al., 1996; Ward et al., 1999). Two female university students majoring in dance studies, both novices to formal ideation tasks, were instructed to co-design a “novel and creative museum” within 15 min. They were encouraged to utilize a whiteboard to facilitate their discussion and illustrate their concepts.

The pair conceptualized a “Moving Museum” defined by two key attributes: (A) artworks displayed without titles to prompt subjective viewer interpretation and active naming, and (B) an exhibition set within the window frames of a moving train, synchronizing the art with the external passing landscape. Both participants reported high levels of satisfaction with this final concept.

**Figure 2:**
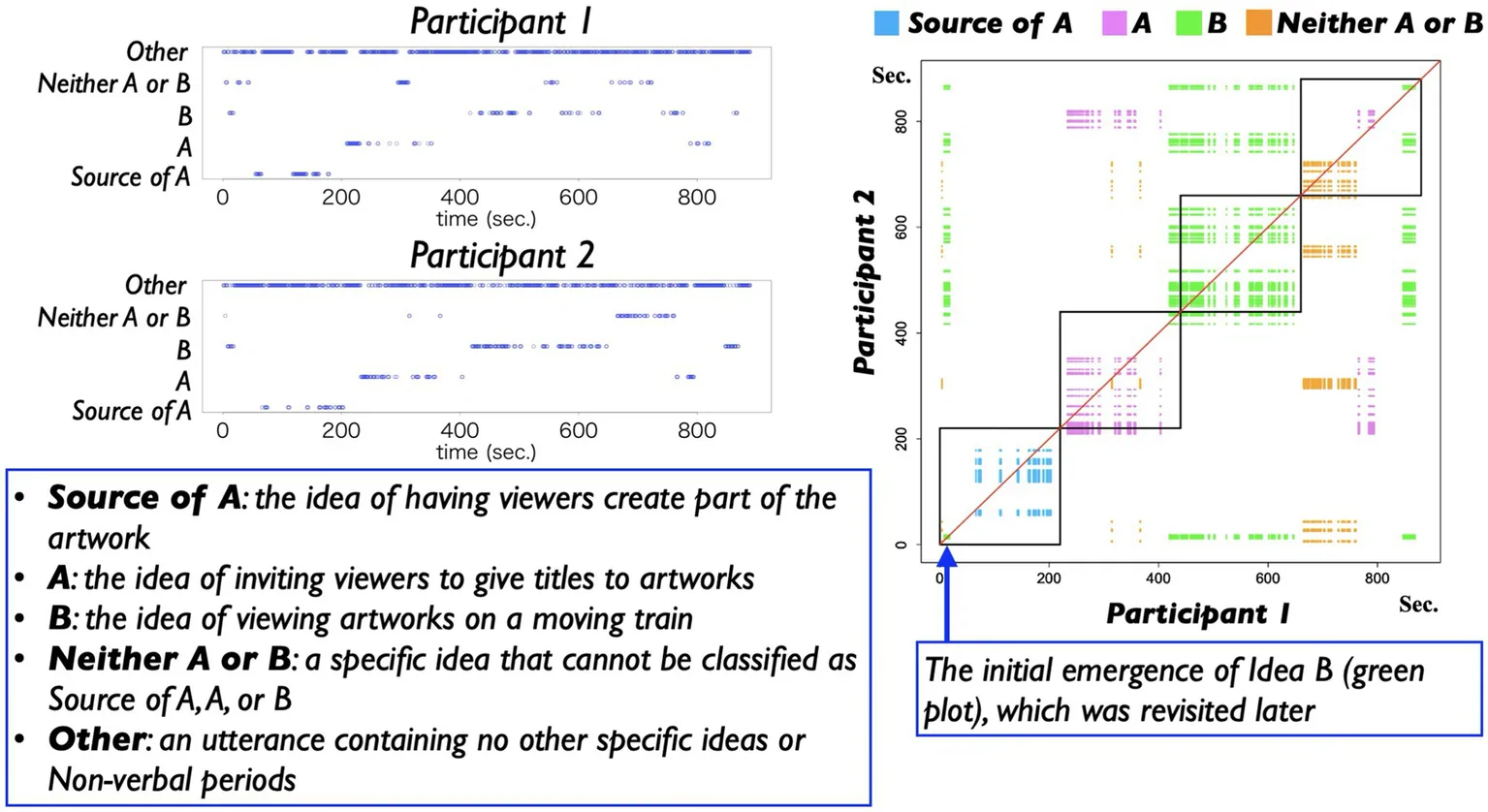
Categorical time series (left) and content-based cross recurrence plot (right) of two participants in the idea generation task.

To analyze this interaction, the utterances were transcribed, and coding categories were generated in a bottom-up manner based on the utterance’s content similarity (Figure 2, left). Next, utterances were coded using ELAN software (Max Planck Institute), with a categorical label assigned to each one-second interval from five distinct categories:

- Source of A: the idea of having viewers create part of the artwork
- A: the idea of inviting viewers to give titles to artworks
- B: the idea of viewing artworks on a moving train
- Neither A or B: a specific idea that cannot be classified as Source of A, A, or B
- Other: an utterance containing no other specific ideas or non-verbal periods

To assess the reliability of the coding scheme, inter-rater agreement was evaluated between two independent coders. The first author and a graduate student specializing in psychology and cognitive science, who was blind to the purpose and results of the study, independently classified all utterances produced by the two participants during the task. The results showed a percent agreement of 85.21% and a Cohen’s *κ* of 0.68. According to the criteria proposed by Landis and Koch (1977), this value indicates substantial agreement, suggesting that the coding scheme achieved an acceptable level of reliability.

A Content-based Cross Recurrence Plot (C^2^RP) was then constructed, mapping Participant 1 on the horizontal axis and Participant 2 on the vertical axis, with matching categories color-coded for visual clarity (Figure 2, right). Segments classified as Other—that is, utterances containing no specific ideas or periods without utterance—were excluded from the C^2^RP and thus were not used to characterize coordination between participants. This choice was motivated by the nature of the idea-generation task, in which the primary focus was on the specific ideas being proposed, discussed, and elaborated by the participants. Further, because the final portion of the recording was partially unclear, the analysis was restricted to the first 880 s of the task. To better capture the dynamic evolution of ideas during the creative process, the analyzed period was then divided into four equal phases of 220 s each. In the present study, the number of phases was determined empirically based on the number of utterance categories while ensuring that each phase captured both sufficient coordination within categories and dynamic transitions between them. Nevertheless, the optimal number of phases may vary depending on the research objectives and the characteristics of the creative task being examined.

The C^2^RP provides a clear qualitative map of the conceptual evolution. The initial phase was dominated by Category 1 (preliminary ideas for Feature A), followed by sustained elaboration of Feature A (Category 2). The discovery of Feature B (Category 3) occurred during the middle-to-late phase while refining the context for Feature A. The session concluded with a refinement period involving auxiliary ideas (Category 4). Interestingly, the plot reveals that elements of Feature B (green clusters) appeared sporadically during the early phase (Figure 2, right; blue arrows and caption). While initially overlooked, these ideas were later reinterpreted and integrated as the participants reflected on the implementation of Feature A. This revisitation—the non-linear reincorporation of earlier thoughts—shows the capacity of C^2^RQA to track iterative creative dynamics.

Furthermore, we examined the direction of interpersonal dynamics. For Feature A (purple clusters), a higher density of points below the diagonal indicates that Participant 2 primarily initiated these ideas, with Participant 1 responding. Conversely, Feature B (green clusters) shows a more balanced distribution, suggesting a bi-directional exchange. This shift implies that while Participant 2 led the development of the initial concept, the subsequent development of the “Moving Museum” was a more symmetrical, collaborative effort, likely facilitated by the shared understanding gained from the idea’s earlier, brief appearance.

Table 1 presents quantitative measures of the frequency and duration of coordination observed in the C^2^RP for each color-coded idea category. These quantitative C^2^RQA metrics are consistent with the observations described above and suggest dynamic changes in coordination across different phases. In this illustrative case, the results demonstrate the potential of the proposed method to visualize and quantify emergence, revisitation, and leader–follower relationships in an ideation environment. It should be noted that, at present, the leader–follower ratio is calculated using the procedure originally proposed in CRQA, namely the ratio of the total coordination in the upper and lower triangles formed by partitioning the recurrence plot along its longest diagonal. An alternative approach would be to exclude periods classified as Other (i.e., utterances containing no specific ideas or non-verbal periods) and compute the ratio using only intervals in which actual ideas were expressed. This would involve removing all recurrence points associated with Other periods from both triangles before calculating the ratio. Whether such an adjustment is appropriate requires careful consideration of the conceptual meaning of the Other category.

**Table 1:** Results of content-based cross recurrence quantification analysis (C^2^RQA) of two participants in the idea generation task.

Phase	%REC	%DET	MaxL	L	ENTR	Leader–follower
Ideas that were the source of feature A (Blue)						
Phase 1	3.44	92.74	6	3.32	1.41	0.83 (above: 1372, below: 289)
Phase 2	0	NA	0	0	NA	NA (above: 0, below: 0)
Phase 3	0	NA	0	0	NA	NA (above: 0, below: 0)
Phase 4	0	NA	0	0	NA	NA (above: 0, below: 0)
Ideas related to feature A (Purple)						
Phase 1	0	NA	0	0	NA	NA (above: 0, below: 0)
Phase 2	2.9	72.08	11	3.05	1.4	0.09 (above: 133, below: 1270)
Phase 3	0	NA	0	0	NA	NA (above: 0, below: 0)
Phase 4	0.35	90.48	4	2.71	0.96	1 (above: 168, below: 0)
Ideas related to feature B (Green)						
Phase 1	0.11	61.11	3	2.2	0.5	0 (above: 0, below: 54)
Phase 2	0.19	71.11	5	3.2	1.33	1 (above: 90, below: 0)
Phase 3	7.62	73.22	8	2.73	1.17	0.29 (above: 1,055, below: 2,616)
Phase 4	0.63	77.78	5	2.51	0.94	1 (above: 306, below: 0)
Ideas not related to feature A or B (Orange)						
Phase 1	0.02	NA	0	0	NA	0 (above: 0, below: 11)
Phase 2	0.16	94.74	2	2	0	0.49 (above: 37, below: 38)
Phase 3	0	NA	0	0	NA	NA (above: 0, below: 0)
Phase 4	1.49	69.72	3	2.18	0.48	0 (above: 0, below: 720)

### 3.2 Experimental dataset 2: Collaborative insight problem-solving

Next, we applied the methodology to a dyadic insight problem-solving task. Insight problems typically require the relaxation of implicit cognitive constraints to reach a “eureka” moment (Ohlsson, 1992; Knoblich et al., 1999).

**Figure 3:**
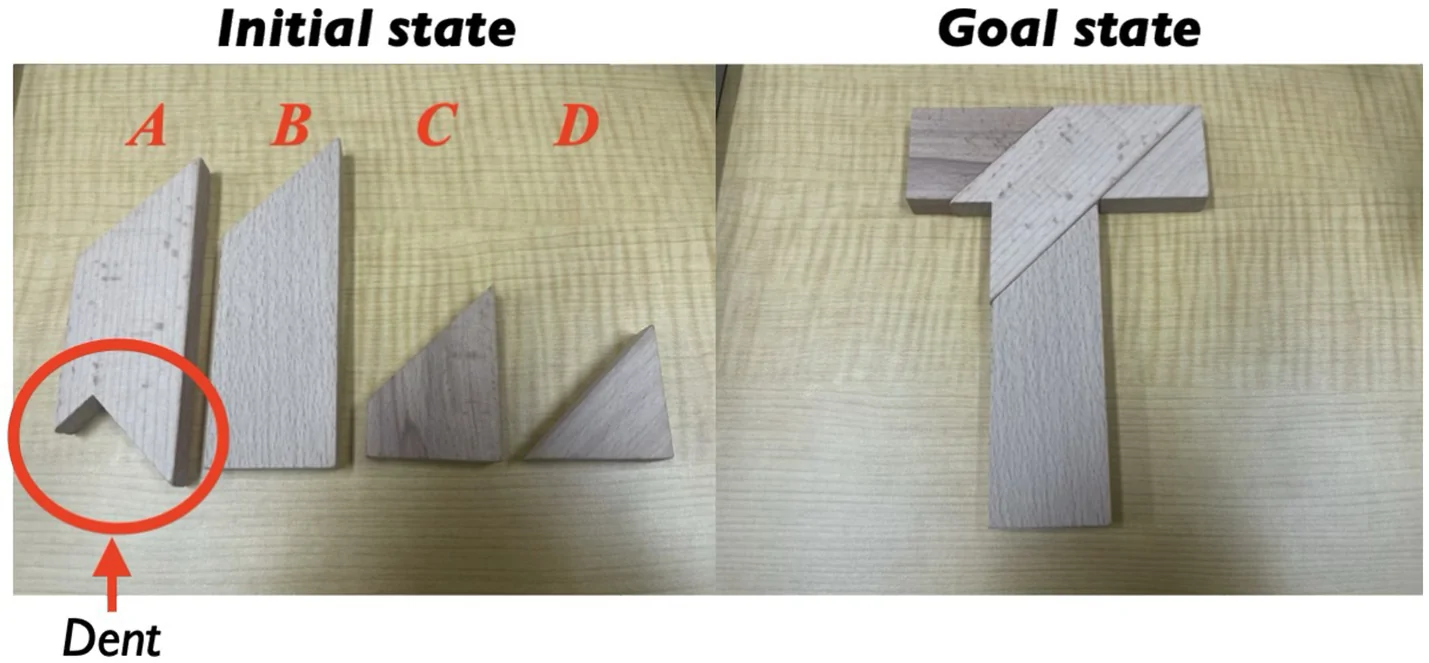
Outline of T-puzzle. Participants combine the four parts to create the letter T.

We utilized the T-puzzle (Figure 3), where the primary obstacles are (1) the tendency to orient pieces only vertically or horizontally (Constraint 1) and (2) the tendency to fill the pentagonal piece’s dent (Constraint 2). Success typically follows the relaxation of these constraints, specifically the diagonal placement of piece A (Suzuki et al., 1999). The same participant pair in the idea generation task was given 25 min to solve the puzzle. They reached the correct solution at 990 s, having only realized the significance of the above diagonal strategy at the end of the task (880 s).

To analyze interactions during the insight problem-solving task, we developed a coding scheme for parts manipulation based on previous studies (Suzuki et al., 1999; Kiyokawa and Ueda, 2007).

Participants’ manipulation behaviors were coded using ELAN software (Max Planck Institute), with each one-second interval assigned to one of four distinct categories: (1) vertical/horizontal orientation (first constrained action), (2) dent-filling (second constrained action), (3) diagonal placement (unconstrained action), and (4) other manipulations. As in dataset 1, inter-rater agreement was assessed to evaluate the reliability of the coding scheme. The first author and a graduate student specializing in psychology and cognitive science, who was blind to the purpose and results of the study, independently classified all parts-manipulation behaviors produced by the two participants during the task. The results showed a percent agreement of 98.25% and a Cohen’s *κ* of 0.97. According to the criteria proposed by Landis and Koch (1977), this value indicates almost perfect agreement, suggesting that the coding scheme achieved a high level of reliability.

Consistent with Dataset 1, segments assigned to the “Others” category were excluded from the C2RP and were therefore not used to characterize interpersonal coordination. The “Others” category included part manipulations that did not correspond to any of the four predefined manipulation categories (e.g., holding a piece vertically or lifting a piece into the air), as well as periods during which no identifiable part manipulation was observed (e.g., holding one’s hands still in the air without touching any pieces or clasping both hands together). It should be noted that the dynamic process of constraint relaxation in the present T-puzzle task was inferred indirectly from the frequency and patterns of participants’ part manipulations. As discussed above, several previous T-puzzle studies have examined behavioral aspects of problem solving through analyses of participants’ actions (Suzuki et al., 1999; Kiyokawa and Ueda, 2007). In light of this prior work, we consider such behavior-based inferences to have a reasonable degree of validity, although they should not be regarded as direct measures of internal cognitive processes.

The 880-s mark—where the diagonal strategy was first verbalized—is indicated by a red dotted cross. Further, to better capture the dynamic evolution of solution strategies throughout the insight problem-solving process, the 990-s task period was divided into three equal phases of 330 s each. As discussed for the idea-generation task, the appropriate number of phases should ultimately be selected based on the research objectives and the nature of the task being examined. In the present study, the number of phases was determined empirically based on the number of behavioral categories defined in previous research (Kiyokawa and Ueda, 2007), while ensuring that each phase captured both sufficient coordination within categories and dynamic transitions between them. Nevertheless, we acknowledge that alternative data-driven strategies for phase selection, such as directly comparing multiple candidate phase numbers (e.g., three to six phases) and selecting or reporting the most appropriate solution, should be explored in future studies.

**Figure 4:**
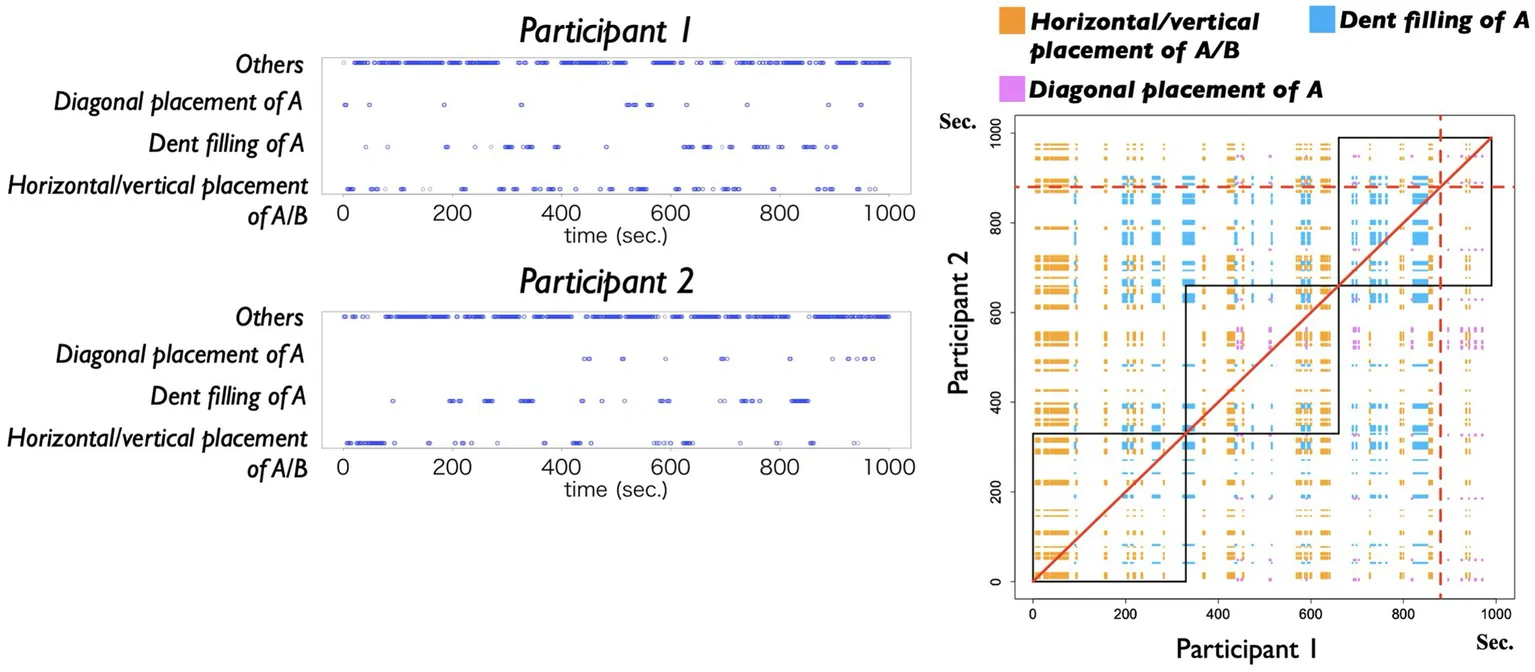
Categorical time series (left) and content-based cross recurrence plot (right) of two participants in the insight problem.

The resulting C^2^RP (Figure 4, left) used yellow for Category 1, blue for Category 2, and purple for Category 3. Qualitative observation of the C^2^RP (Figure 4, right) shows that Phase 1 was dominated by constraint-driven behaviors (Categories 1 and 2). Although Participant 1 independently explored diagonal placements early on, these attempts were not initially reciprocated or recognized as significant by Participant 2 (Figure 4, left). In Phase 2, joint diagonal manipulations (purple plots) began to appear intermittently even before explicit verbal recognition. By Phase 3, constraint-based actions decreased as the diagonal strategy became dominant.

Leader–follower analysis showed that in Phase 2, diagonal manipulations appeared exclusively above the diagonal, indicating that Participant 1 led these actions. However, after the strategy was explicitly recognized in Phase 3, the coordination became bidirectional and reciprocal. This transition from asymmetrical to symmetrical interaction demonstrates C^2^RQA’s sensitivity to evolving interpersonal dynamics.

Table 2 presents quantitative measures of the frequency and duration of coordination observed in the C^2^RP for each color-coded category of parts manipulation. The quantitative C^2^RQA data are consistent with these observations, suggesting a decline in constraint-based coordination and an increase in reciprocal, unconstrained behavior as the solution emerged. Although broader sampling is needed, this case illustrates the potential of the method for quantifying constraint relaxation, strategy shifts, and changes in leader–follower dynamics during collaborative problem-solving. It should be noted that the leader–follower ratio was computed using the procedure adopted in previous studies and dataset 1, namely the ratio of the total coordination in the upper and lower triangles obtained by partitioning the recurrence plot along its longest diagonal.

**Table 2:** Results of content-based cross recurrence quantification analysis (C^2^RQA) of two participants in the insight problem.

Phase	%REC	%DET	MaxL	L	ENTR	Leader–follower
Placing part A/B vertically or horizontally (Orange)						
Phase 1	4.72	92.66	13	4.94	2.13	0.29 (above: 1498, below: 3623)
Phase 2	3.99	92.13	16	3.62	1.67	0.57 (above: 2466, below: 1873)
Phase 3	0.84	77.78	8	2.83	1.24	0.49 (above: 451, below: 463)
Filling the dent in part A (Blue)						
Phase 1	1.18	92.02	15	3.79	1.63	0.9 (above: 1161, below: 128)
Phase 2	1.47	93.81	12	4.35	1.97	0.29 (above: 455, below: 1,141)
Phase 3	3.74	95.11	13	4.39	1.98	0.22 (above: 906, below: 3,154)
Placing part A diagonally (Purple)						
Phase 1	0	NA	0	0	NA	NA (above: 0, below: 0)
Phase 2	0.35	91.53	5	2.98	1.27	0.74 (above: 280, below: 98)
Phase 3	0.25	82.22	4	2.49	0.87	0.66 (above: 177, below: 91)

### 3.3 Field dataset: collaborative drawing activity in an art workshop

**Figure 5:**
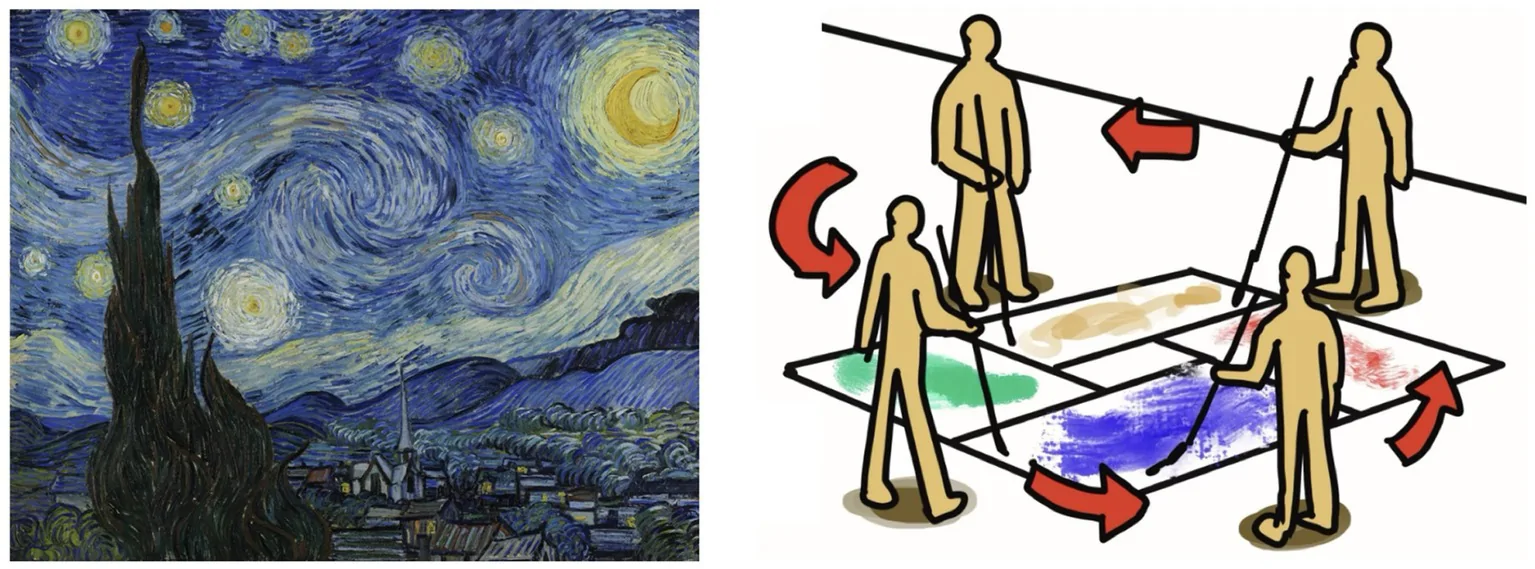
Artworks selected for appreciation and creation in the art workshop (left: Vincent van Gogh. *The Starry Night* (1889). Collection of The Museum of Modern Art, New York. Public domain image. Source: Wikimedia Commons). Outline of the collective drawing activities in the art workshop (right).

As a third case, the proposed analytical framework was applied to a drawing activity conducted within an art workshop involving groups of four to five participants. This workshop was designed to foster collaborative creativity among business professionals, aligning with the growing emphasis on STEAM and 21st-century education (e.g., OECD, 2018; Perignat and Katz-Buonincontro, 2019; Shimizu et al., 2021). The program was co-developed and executed with professional artists experienced in museum-based educational engagement. The workshop employed an iterative structure where participants alternated between collective art appreciation (Figure 5, left) and collective drawing inspired by those artworks. This design aimed to facilitate mutual recognition of unique aesthetic perspectives of appreciation and creation, and to expand individual creative boundaries through inspiration.

The focal activity was a 30-min group drawing session held at the final phase of the workshop. Four groups, each comprising four to five participants (e.g., advertising professionals; mean age: 33.91 years, range: 21–42, 9 females, 9 males), engaged in the task. Participants’ individual drawings from previous sessions were integrated into a single large canvas, around which the group members were positioned (Figure 5, right). To encourage the overwriting and reinterpretation of both their own and others’ drawings, participants were instructed to rotate their physical positions every 5 min in a counter-clockwise direction. This resulted in four distinct drawing phases (the initial position plus three rotations). Facilitators explicitly encouraged participants to treat the canvas as a collective output rather than a collection of individual works. The entire process was recorded via video camera for subsequent analysis.

**Figure 6:**
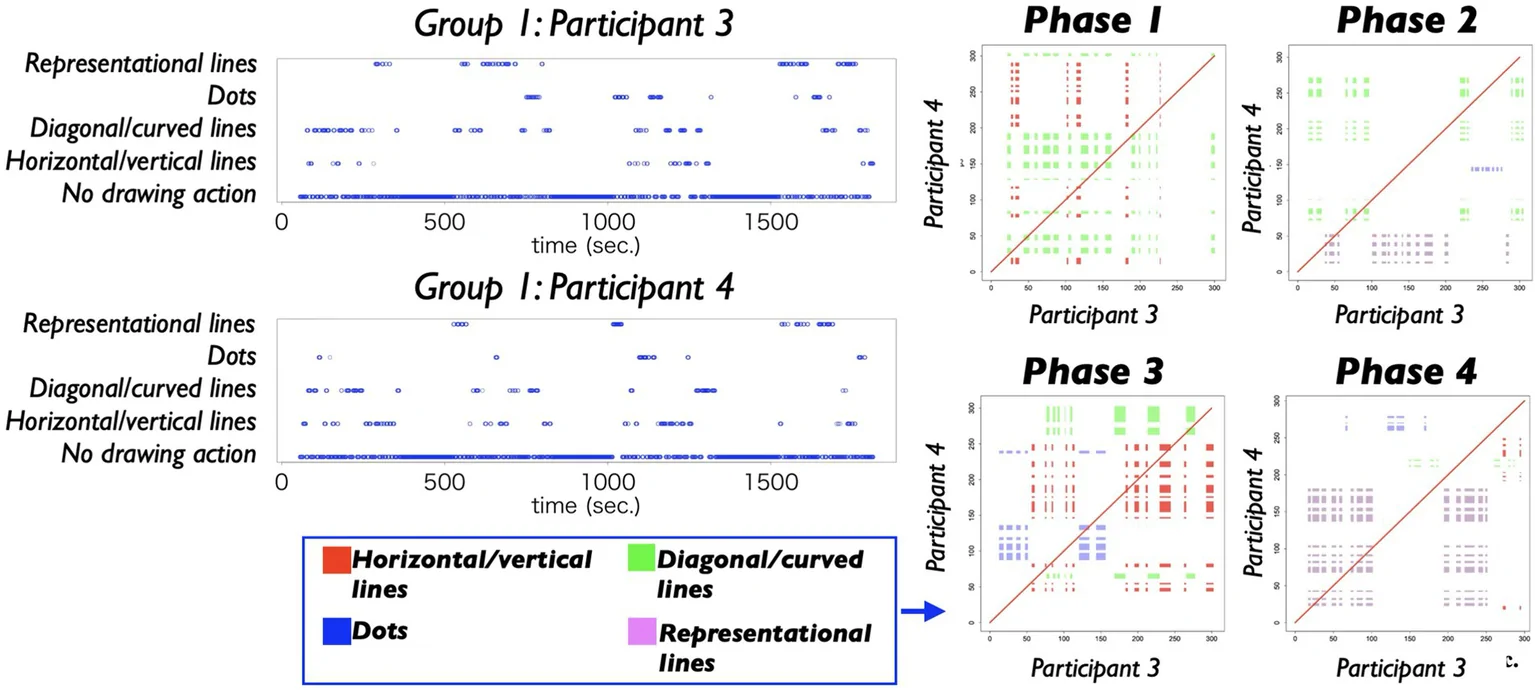
Categorical time series (left) and Content-based Cross Recurrence Plot (right) of two representative participants in the drawing activities in the art workshop. Participants 3 and 4 in Group 1 were selected as a representative example because they exhibited particularly pronounced changes across phases. However, as can be seen from both the mean values and the raincloud plots shown in Figure 7, similar dynamic phase-to-phase changes were also observed in other participant pairs across the remaining groups.

Analysis began with the systematic classification of stroke types captured in the video. Five distinct categories were identified using a bottom-up approach based primarily on the most frequently observed behaviors: (1) no drawing action, (2) horizontal/vertical lines, (3) diagonal/curved lines, (4) dots, and (5) representational lines (depicting specific objects) (Figure 6, left). Coding was performed in ELAN (Max Planck Institute) at the level of individual strokes (from contact to release on the canvas), with a single category label assigned to each one-second interval from the categories described above. It should be noted that the three categories—horizontal/vertical, diagonal/curved, and dots—were mutually exclusive. In contrast, representational lines occasionally overlapped with the other categories. In the present study, such drawings were interpreted as being primarily intended to depict a specific representation and were therefore classified exclusively into the representational lines category. This decision was made because the proposed analytical framework required a categorical time series in which each time point was assigned to a single category.

C^2^RP was then applied to these categorical time-series data to evaluate participants’ interaction. Consistent with datasets 1 and 2, segments assigned to the no drawing action category, representing periods in which no specific drawing behavior was observed, were excluded from the C^2^RP and were therefore not used to characterize interpersonal coordination. As noted above, this work consisted of four drawing sessions, with a brief break inserted after every 5 min of drawing activity. Therefore, each 5-min session was defined as a separate phase.

We first examined whether participants within the same group influenced each other’s stroke variations. Figure 6 (right) illustrates the C^2^RP for a representative pair (Participant 3 and 4) from Group 1. During Phase 1, interactions were dominated by the exchange of structural lines (horizontal/vertical: red plot and diagonal/curved: green plot). By Phases 2 and 4, however, there was a marked increase in the reciprocal use of representational lines (purple plot). Phase 3 exhibited a diverse mixture of all stroke types, including dots (blue plot). These patterns suggest that participants exert direct and active influence on one another’s drawing behaviors, with the nature of this interaction evolving dynamically as the session progresses.

**Figure 7:**
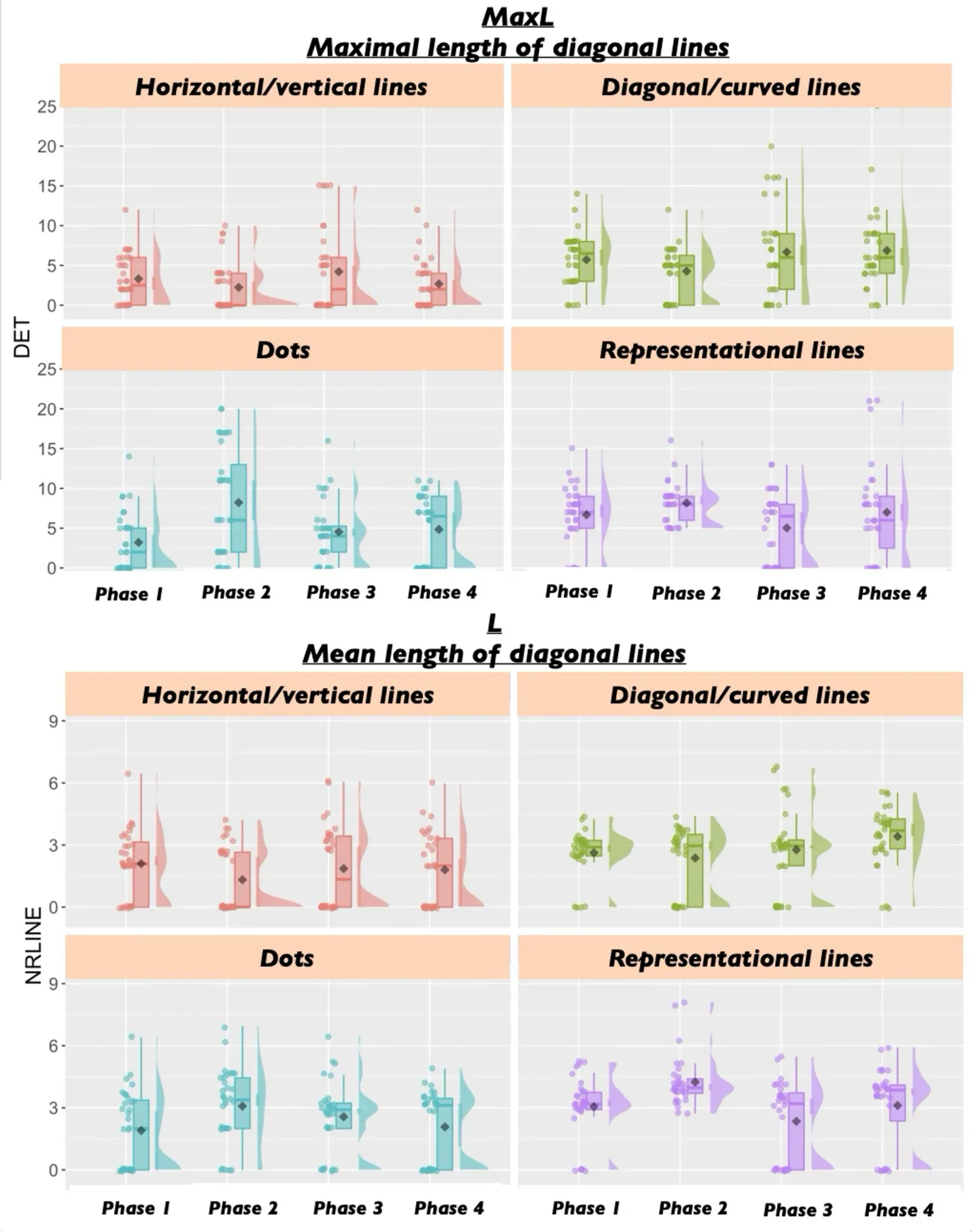
Results of content-based cross recurrence quantification analysis (C^2^RQA) of all participant pairs in the drawing activities in the art workshop.

Aggregated results across all pairs are shown in Figure 7. To examine these patterns statistically, we conducted a Linear Mixed Model (LMM) analysis, treating stroke type and phase as fixed effects and group, pair, and individual as random effects (Table 3). Random effects were specified as random intercepts only; random slopes were not included in the model. The LMM was specified as:


$$\begin{array}{l} C^{2}\mathrm{RQA}\;\text{Metrics}\sim {\text{StrokeType}}^{*}\;\text{Phase}+(1|\text{Group})+\\ \left(1|\text{Pair})+(1|\text{Participant}\right) \end{array}$$

**Table 3:** Results of linear mixed modeling of C^2^RQA metrics of whole participants in the drawing activities in the art workshop.

Fixed effects	*β*	SE	*p*-value
%REC			
Representational lines	0.014	0.0029	>0.001
Phase 2	−0.00045	0.0029	0.88
Representational lines * Phase 2	0.02	0.0059	0.00058
%DET			
Diagonal/curved lines	0.23	0.046	>0.001
Dots	0.02	0.051	0.7
Phase 2	−0.12	0.053	0.029
Representational lines	0.17	0.051	0.00093
Dots * Phase 2	0.35	0.092	0.00017
Representational lines * Phase 2	0.33	0.092	0.0003
MaxL			
Diagonal/curved lines	2.41	1.17	0.041
Phase 2	−1.06	1.17	0.37
Phase 3	0.91	1.17	0.44
Phase 4	−0.62	1.17	0.59
Dots	−0.094	1.17	0.94
Representational lines	3.38	1.17	>0.001
Diagonal/curved lines * Phase 2	−0.38	1.66	0.82
Diagonal/curved lines * Phase 3	0.062	1.66	0.97
Diagonal/curved lines * Phase 4	1.78	1.66	0.28
Dots * Phase 2	6.06	1.66	>0.001
Dots * Phase 3	0.41	1.66	0.81
Dots * Phase 4	2.25	1.66	0.18
Representational lines * Phase 2	5.09	1.66	>0.001
Representational lines * Phase 3	−2.56	1.66	0.12
Representational lines * Phase 4	1.53	1.66	0.36
L			
Diagonal/curved lines	1.09	0.21	>0.001
Dots	0.25	0.24	0.29
Phase 2	−0.63	0.25	0.011
Representational lines	0.91	0.24	>0.001
Dots * Phase 2	1.53	0.43	>0.001
Representational lines * Phase 2	2.26	0.43	>0.001

Stroke types and phases are included as the fixed effects, and group, pair, participant as the random effects.

Linear mixed-effects models were fitted using restricted maximum likelihood (REML) estimation. Model selection was conducted using a backward elimination procedure. Starting from the full model including all fixed effects and interactions, non-significant terms were sequentially removed based on likelihood ratio tests. For each C^2^RQA metric, *post hoc* pairwise comparisons were adjusted using the Bonferroni correction to control for multiple testing.

**Figure 8:**
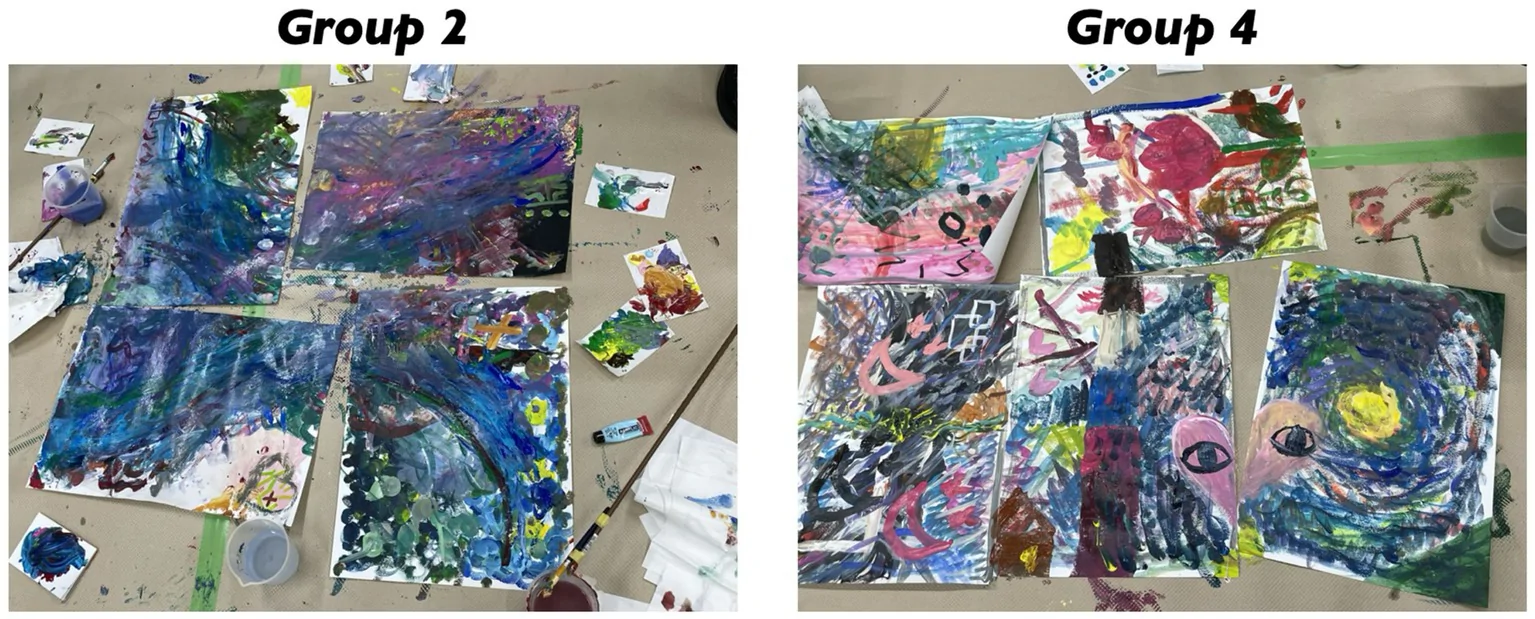
Representative examples of the artworks created through the collective drawing activities in the art workshop.

The results indicated that diagonal/curved lines and representational lines were generally more prevalent than the other drawing categories throughout the session. Phase 2 was characterized by a temporary decline in the overall frequency of reciprocal drawing coordination. Despite this decline, representational and diagonal/curved lines remained relatively prominent. Given that the completed artworks contained a complex mixture of figurative and abstract elements (Figure 8), it is noteworthy that coordination involving representational lines—those depicting specific objects or imagery—increased during Phase 2, decreased thereafter, and then increased again in Phase 4. This temporal pattern suggests that participants repeatedly shifted between more concrete, representational forms of expression and more abstract forms of drawing during the collaborative creative process.

It remains possible that the observed interaction in stroke types was not a result of mutual interpersonal influence, but rather emerged by chance as each participant drew independently according to their personal habits. To address this possibility and isolate the effect of genuine interaction, we constructed Virtual pairs. Virtual pairs were constructed by replacing one participant’s data in a real pair with data from the same participant obtained in a different phase. For example, if a real pair consisted of Participant 1 (Phase 1) and Participant 2 (Phase 1), a virtual pair was created by pairing Participant 1 (Phase 1) with Participant 2 (Phase 2). To generate virtual pairs, substitutions were performed for all alternative phases. In the above example, virtual pairs were created by pairing Participant 1 (Phase 1) with Participant 2 from Phases 2, 3, and 4, respectively, and the resulting values were extracted for comparison with the real pair. This procedure was intended to control for individual drawing tendencies while isolating patterns that emerged through actual interpersonal interaction. Since all phases were conducted for the same duration (5 min), no adjustment for time-series length was necessary.

The results indicated that the Real pairs exhibited a significantly higher degree of reciprocal use of strokes for diagonal and curved lines compared to the Virtual pairs. Furthermore, for dots and representational lines, the Real pairs showed a markedly stronger tendency to co-occur during Phase 2 (refer to the Supplementary material for detailed results). These findings provide empirical evidence that the observed drawing-style matching was not an artifact of individual drawing styles, but was indeed a manifestation of active, real-time social interaction between participants.

Finally, we analyzed the directionality of these interactions. By dividing the C^2^RP along the main diagonal, we calculated the relative proportion of leading versus following for each participant. Specifically, we tracked the extent to which the participant who tended to lead in Phase 1 continued to lead in subsequent phases using the following formula:


$$\begin{array}{l} \text{Lead Ratio of Phase}\;1\;\text{Leader}=(\begin{array}{l} \text{Plots initiated}\;\mathrm{by}\;\\ \text{the Phase}\;1\;\text{leader} \end{array})/\\ \left(\text{Total plots of the pair}\right) \end{array}$$

**Figure 9:**
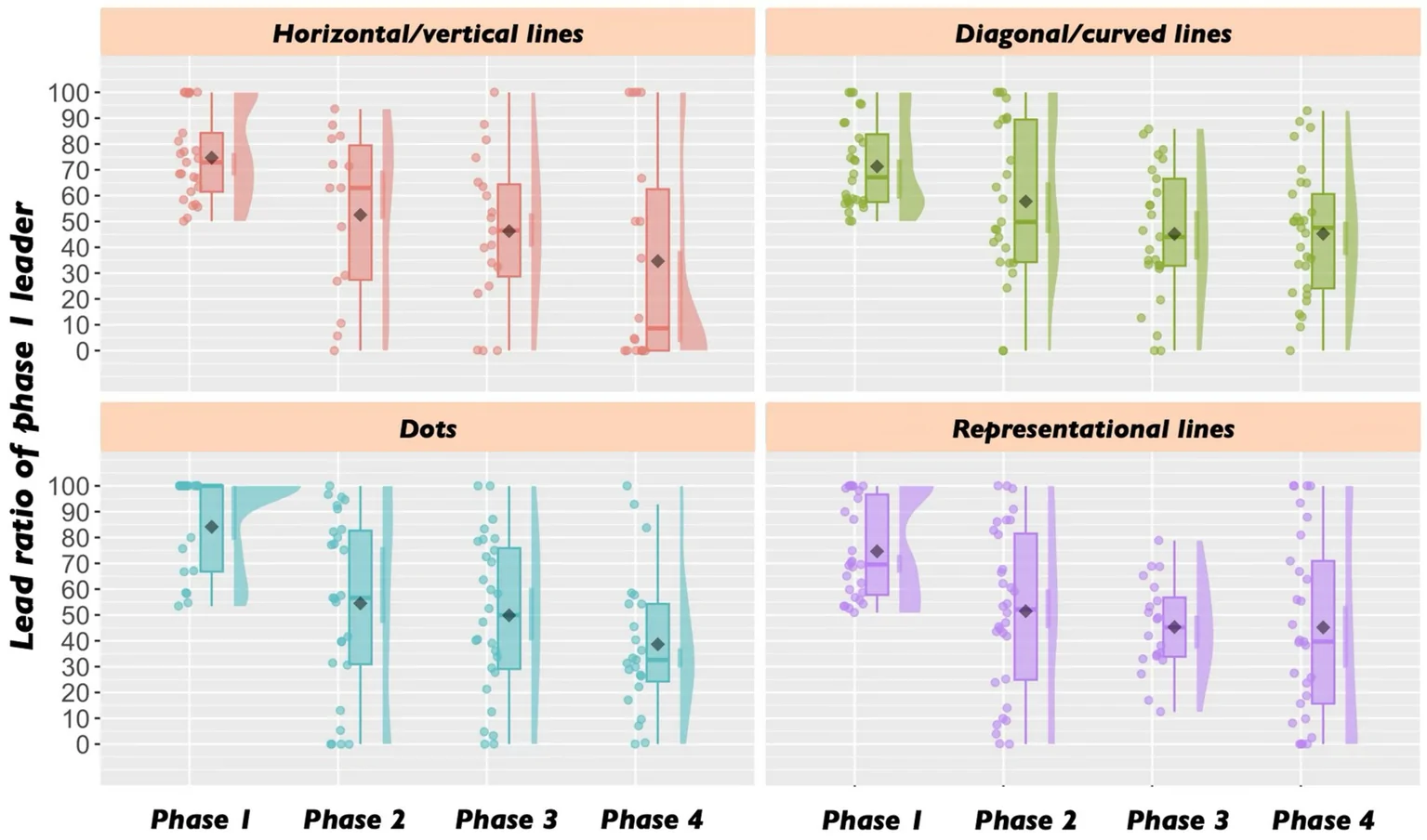
Results of the lead ratio of the phase 1 leader in the drawing activities in the art workshop.

As shown in Figure 9, Phase 1 was characterized by highly asymmetrical interactions, with one participant leading the drawing style 60–100% of the time. However, Phases 2 through 4 showed a progressive shift toward bidirectional interaction and increased variance between pairs. The Coefficient of Variation (CV) for vertical/horizontal lines rose from 0.23 (Phase 1), 0.62 (Phase 2), 0.63 (Phase 3), 1.20 (Phase 4), and for representational lines from 0.25, 0.61, 0.39, 0.78. Bootstrap-calculated 95% confidence intervals confirmed that Phase 1 CV values were significantly lower than in subsequent phases (refer to the Supplementary material for detailed results). This indicates that as the drawing activity progressed, the rigid leader–follower roles dissolved into more reciprocal and unpredictable exchanges. This transition toward symmetrical coordination matches the dynamics observed in ideation and insight tasks, suggesting a fundamental principle of collaborative creativity.

## 4 Discussion

The present study introduced C^2^RQA as a useful framework for visualizing and quantifying collective creative processes. By applying this method across three distinct experimental and field datasets—idea generation, insight problem-solving, and drawing session in an art workshop—we illustrated its applicability to capture several key dimensions of collaborative creative processes. Specifically, the results suggest that C^2^RQA can track the emergence and revisitation of ideas, capture the overall structure of interaction reflected in participants’ utterances and actions, and reveal directional lead–lag dynamics between collaborators. Taken together, these findings suggest that the proposed analytical framework has the potential to capture aspects of the Emergence and Revisitation dimension, while also providing an initial basis for examining the Representation and Embodiment dimension introduced in the theoretical framework.

On the other hand, the third extension of the proposed framework—the analysis of interplay across different modalities—has not yet been empirically examined in the present study. Accordingly, our discussion of this aspect remains at the level of a conceptual and methodological possibility. Whether the proposed framework can effectively capture the dimension of Conscious and Subconscious Interplay introduced in the introduction, therefore, remains an open question and warrants further careful investigation. While the current study focused on individual modalities, future inquiries could employ the Joint Recurrence Plots approach (JRP, Marwan, 2008) and develop an integrated cross-media recurrence technique (Content-based JRP) to directly analyze synchronous patterns across disparate media, such as the co-occurrence of verbalizations and physical actions. These extensions are expected to facilitate the examination of interactional processes that are not fully verbalized or consciously articulated, particularly those manifested through participants’ actions and embodied behaviors.

Regarding the limitations and future outlook of this study, several points warrant consideration. As this study prioritized validating the C^2^RQA framework through a single-pair case study in two experimental studies or a limited number of participants in a field study, the generalizability of the findings remains to be established through larger-scale statistical or Bayesian testing. Nevertheless, the successful quantification of the collaborative process underscores the method’s potential. Extending this analysis to a broader sample would permit rigorous comparisons across different temporal phases or experimental conditions, as the field dataset showed. Theoretically, future research could integrate C^2^RQA metrics with product-level assessments—such as the Consensual Assessment Technique (Amabile et al., 1986) or novelty/utility ratings (Finke et al., 1996)—within a Linear Mixed Model framework. Such an approach would clarify how specific process dynamics correlate with the quality of creative outcomes, a relationship we intend to explore in forthcoming studies.

A second limitation concerns the fact that all three datasets examined in the present study consisted of categorical data. Consequently, the proposed framework has not yet been empirically demonstrated using binary or continuous data. Future studies should therefore examine its applicability to a broader range of data types, particularly continuous time series, as well as to a wider variety of research contexts beyond collaborative creativity. One possible direction is to extend the framework to interpersonal coordination in other domains. For example, we are currently applying aspects of the proposed framework to investigate directional roles in Latin dance interaction (Matsuoka-Hatsuda and Shimizu, under review), although this work does not involve a creative task. Nevertheless, the applicability of the proposed framework to continuous data remains to be systematically evaluated.

A third limitation concerns the application of the proposed framework to groups consisting of more than two participants. The current framework is primarily designed to capture dyadic interactions. As illustrated by dataset 3, extending the method to groups of three or more participants represents an important direction for future research. For three-person groups, one possible approach would be to adapt methods proposed in previous studies on Multidimensional RQA (Wallot et al., 2016) by representing each participant’s time series within a three-dimensional space and visualizing points of coordination among them. For groups of four or more participants, another possibility would be to extend approaches from Joint RQA (Marwan, 2008) by superimposing recurrence plots across all participant pairs and identifying interaction states shared across the group.

An additional and potentially fruitful direction would be to incorporate variability in coordination strength within groups as a research question in its own right. For example, future studies could compare the most strongly coordinated pairs, the weakest pairs, and pairs exhibiting intermediate levels of coordination within the same group. Such an approach may provide a more ecologically valid perspective on collaborative interaction and could also be extended to asymmetric interaction structures, such as two-against-one configurations. Taken together, these possibilities highlight the extensibility of the proposed framework, while also underscoring the need for careful and systematic methodological development in future work.

Moreover, the proposed C^2^RQA framework holds significant potential for application in ecological, real-world settings, including corporate brainstorming, interdisciplinary artistic collaborations, and educational creativity workshops, as demonstrated by our field research data. By focusing on multimodal inputs such as verbalization and action, researchers can quantitatively examine the granular evolution of ideas and artifacts in these real-world contexts. For instance, in the performing or visual arts, classifying specific gestures or brushstrokes and their verbalization, and visualizing their interpersonal correspondence through C^2^RQA could reveal the multi-modal shifts and dynamics that critically define artistic interaction.

In conclusion, we plan to further examine the applicability of the proposed analytical framework by investigating diverse creative processes and products across a larger cohort of participant pairs.

## Data Availability

The raw data supporting the conclusions of this article will be made available by the authors, without undue reservation. However, the original video and audio recordings of participants performing the tasks cannot be made publicly available, due to anonymity restrictions required by the ethics approval.
