# Supplementary material for: C2RQA as the quantification method of the dynamics of collaborative creativity
Source: Front Psychol. 2026 Sep 1;17:1825028. doi: 10.3389/fpsyg.2026.1825028 (PMC13580397; doi:10.3389/fpsyg.2026.1825028)
Supplement: Supplementary file 1 [file Data_Sheet_1.PDF]

Supplement materials

Figure A: Real-Virtual comparison

RR

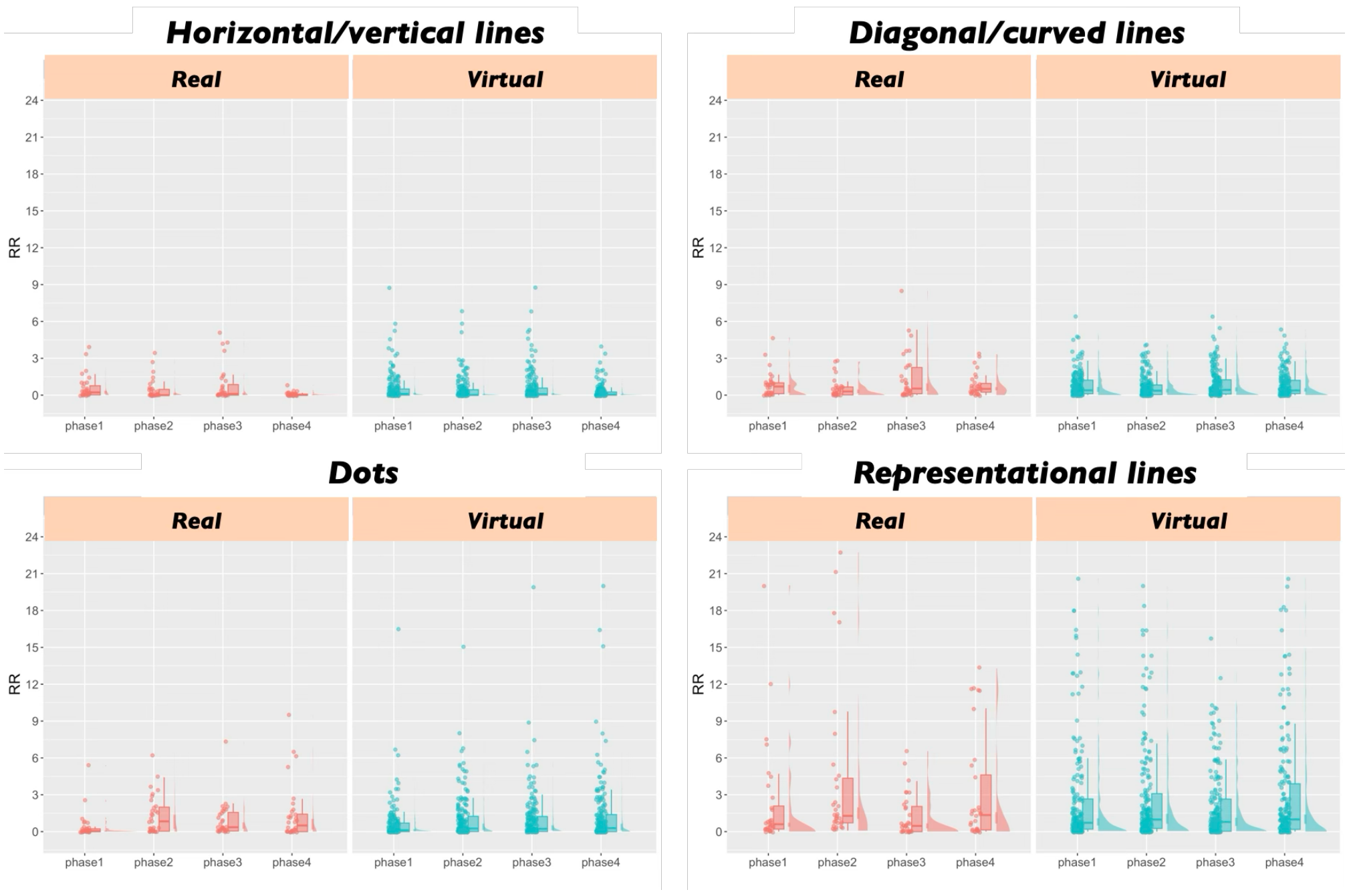

DET

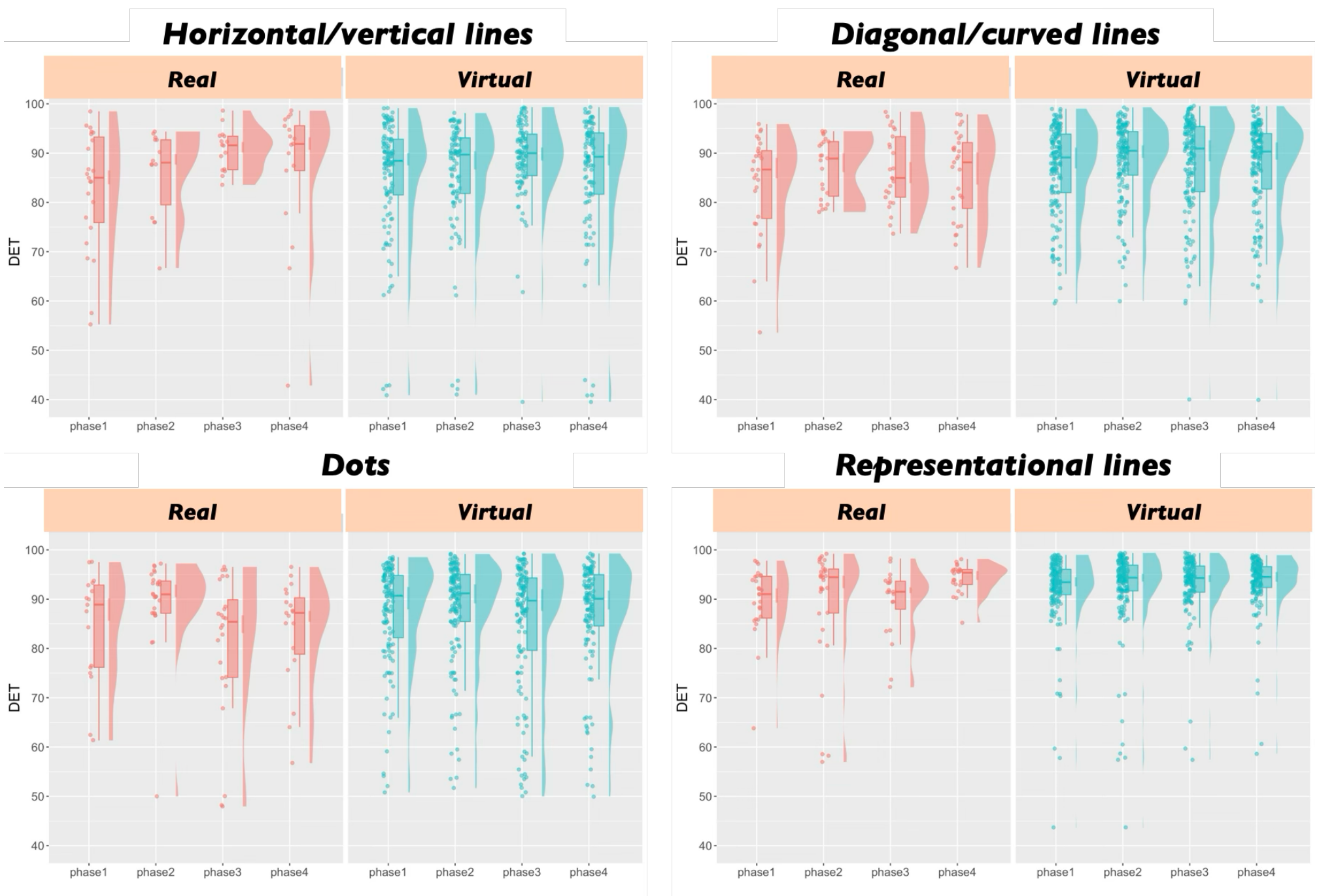

MaxL

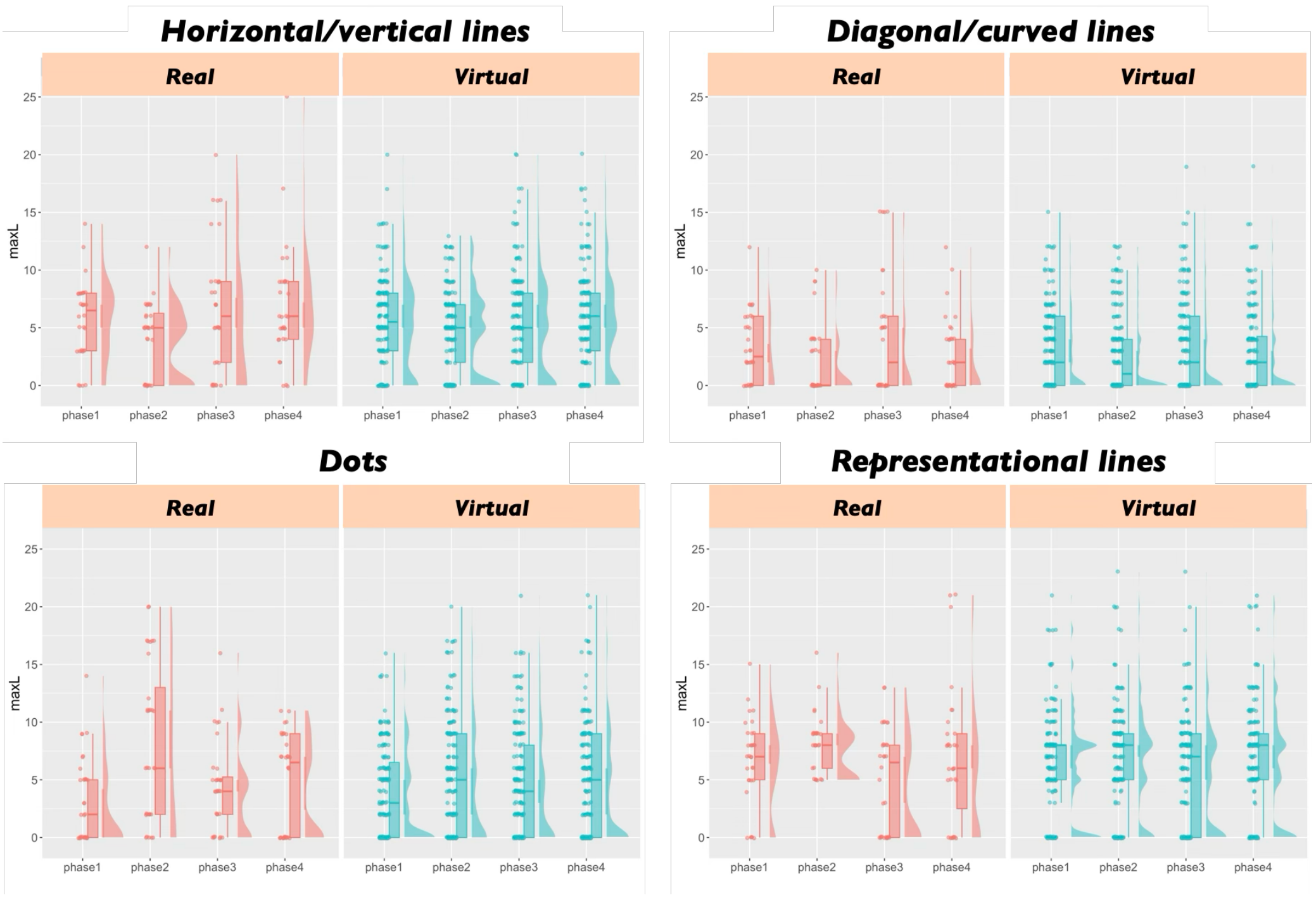

Horizontal/vertical lines
Diagonal/curved lines
Dots
Representational lines
Real
Virtual
maxL
phase1
phase2
phase3
phase4

L

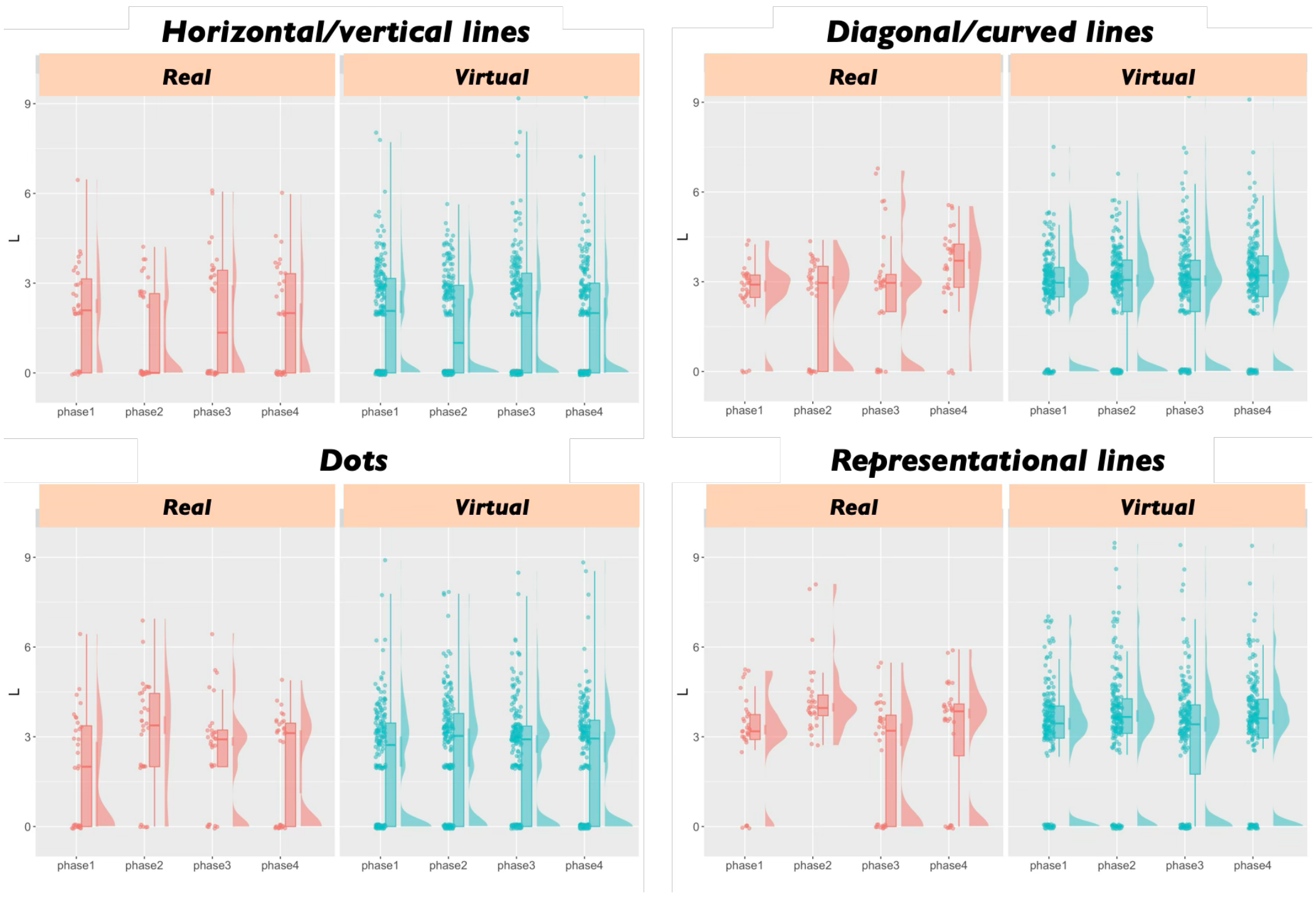

Horizontal/vertical lines
Diagonal/curved lines
Dots
Representational lines
Real
Virtual
L
phase1
phase2
phase3
phase4
