# Supplementary material for: C2RQA as the quantification method of the dynamics of collaborative creativity
Source: Front Psychol. 2026 Sep 1;17:1825028. doi: 10.3389/fpsyg.2026.1825028 (PMC13580397; doi:10.3389/fpsyg.2026.1825028)
Supplement: Supplementary file 2 [file Data_Sheet_2.PDF]

Supplement materials

Figure B: LMM for the lead ratio of the phase 1 leader

	β	*SE*	*p-value*
Diagonal/curved lines	-3.26	7.67	0.67
Phase 2	-22.24	9.31	0.02
Phase 3	-28.58	8.47	0
Phase 4	-39.68	8.62	> .001
Dots	9.78	8.62	0.26
Representational lines	0.14	7.73	0.99
Diagonal/curved lines * Phase 2	8.52	12.05	0.48
Diagonal/curved lines * Phase 3	2.46	11.42	0.83
Diagonal/curved lines * Phase 4	13.62	11.35	0.23
Dots * Phase 2	-7.86	12.58	0.53
Dots * Phase 3	-5.9	11.94	0.62
Dots * Phase 4	-6.24	12.32	0.61
Representational lines * Phase 2	-1	11.82	0.93
Representational lines * Phase 3	-1.11	11.8	0.93
Representational lines * Phase 4	10.2	11.38	0.37

Bootstrap calculation for CV of the lead ratio of the phase 1 leader

Horizontal/vertical lines

Condition 1	*Condition 2*	*p_value*
Phase 1	Phase 2	>.001
Phase 1	Phase 3	>.001
Phase 1	Phase 4	>.001
Phase 2	Phase 3	0.92
Phase 2	Phase 4	0.026
Phase 3	Phase 4	>.001

Diagonal/curved lines

Condition 1	*Condition 2*	*p_value*
Phase 1	Phase 2	>.001
Phase 1	Phase 3	>.001
Phase 1	Phase 4	>.001
Phase 2	Phase 3	0.75
Phase 2	Phase 4	0.92
Phase 3	Phase 4	0.87

Dots

Condition 1	*Condition 2*	*p_value*
Phase 1	Phase 2	>.001
Phase 1	Phase 3	>.001
Phase 1	Phase 4	>.001
Phase 2	Phase 3	0.88
Phase 2	Phase 4	0.73
Phase 3	Phase 4	0.59

Representational lines

Condition 1	*Condition 2*	*p_value*
Phase 1	Phase 2	>.001
Phase 1	Phase 3	0.016
Phase 1	Phase 4	>.001
Phase 2	Phase 3	0.072
Phase 2	Phase 4	0.22
Phase 3	Phase 4	>.001
