# Supplementary material for: C2RQA as the quantification method of the dynamics of collaborative creativity
Source: Front Psychol. 2026 Sep 1;17:1825028. doi: 10.3389/fpsyg.2026.1825028 (PMC13580397; doi:10.3389/fpsyg.2026.1825028)
Supplement: Supplementary file 3 [file Data_Sheet_3.PDF]

Supplement materials

Figure C: Content-based Cross-Recurrence Plot for the insight problem-solving task using four-phase segmentation

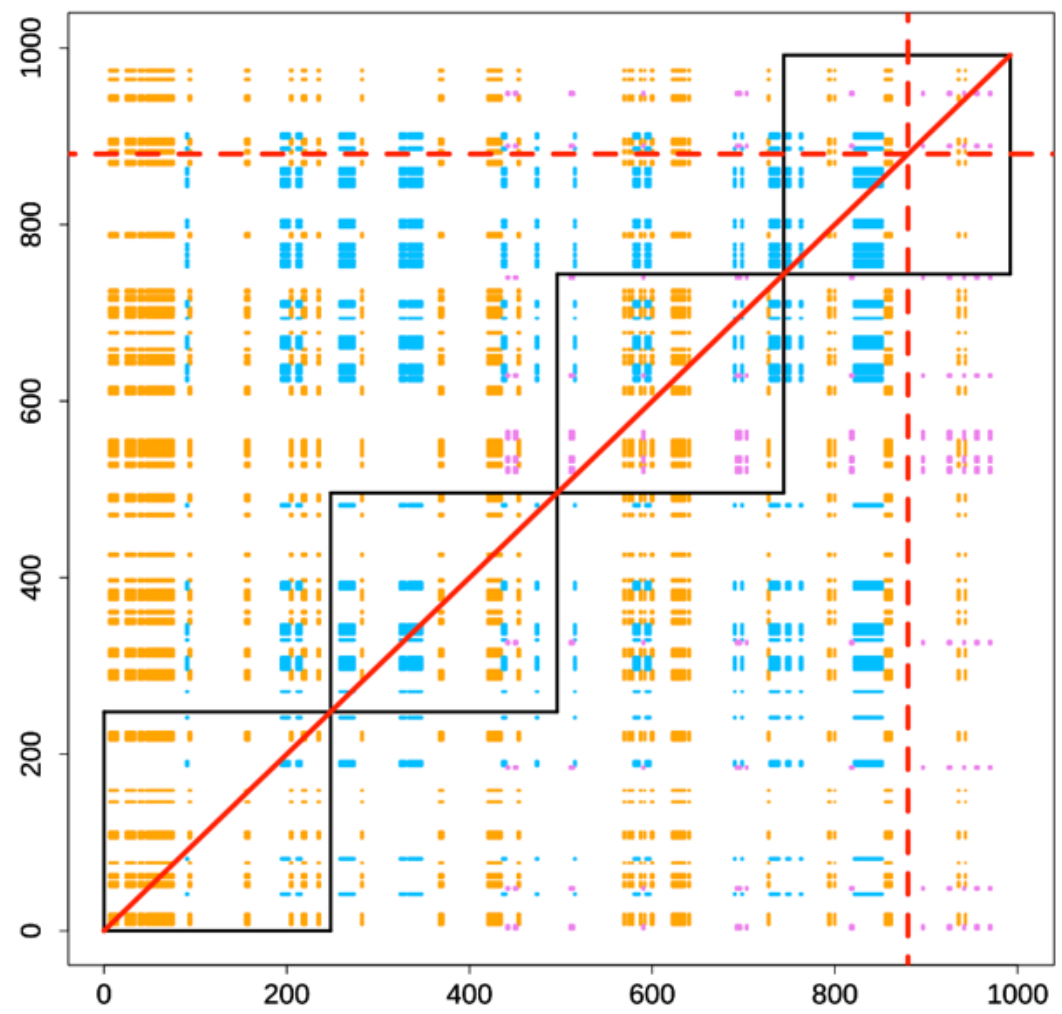

Table A. Results of Content-based Cross Recurrence Quantification Analysis (C^2RQA) for the insight problem-solving task using four-phase segmentation

Placing part A/B vertically or horizontally (Orange)

	%REC	%DET	MaxL	L	ENTR	Leader-Follower
phase_1	5.63	90.74	13	5.01	2.09	0.27 (above: 925, below: 2527)
phase_2	2.45	95.23	12	4.01	1.8	0.65 (above: 963, below: 529)
phase_3	3.38	88.3	15	3.05	1.22	0.54 (above: 1110, below: 956)
phase_4	0.76	77.63	7	2.98	1.35	0.60 (above: 274, below: 186)

Filling the dent in part A (Blue)

	%REC	%DET	MaxL	L	ENTR	Leader-Follower
phase_1	0.37	89.47	6	2.68	0.99	0.86 (above: 196, below: 32)
phase_2	3.28	95.14	15	5.18	2.2	0.18 (above: 371, below: 1636)
phase_3	1.85	93.16	11	3.93	1.74	0.75 (above: 853, below: 285)
phase_4	3.45	97.17	10	4.39	1.81	0.05 (above: 96, below: 2015)

Placing part A diagonally (Purple)

	%REC	%DET	MaxL	L	ENTR	Leader-Follower
phase_1	0	NA	0	0	NA	NA (above: 0, below: 0)
phase_2	0.05	85.71	4	2.67	0.96	1 (above: 28, below: 0)
phase_3	0.73	91.11	6	3.04	1.32	0.33 (above: 149, below: 300)
phase_4	0.22	81.95	4	2.48	0.86	0.91 (above: 121, below: 12)
